# Bolton’s ratio variations in Angle’s Class I, Class II and Class III malocclusions: An observational study

**DOI:** 10.4317/jced.62591

**Published:** 2025-03-01

**Authors:** Pinaki Roy, Poulomi Roy, Sourav Koley, Subhasis Sheet

**Affiliations:** 1 Associate Professor, Dept. of Orthodontics and Dentofacial Orthopaedics, Burdwan Dental College & Hospital, Burdwan; 2 Assistant Professor, Dept. of Orthodontics and Dentofacial Orthopaedics, Burdwan Dental College & Hospital, Burdwan; 3 Assistant Professor, Dept. of Dentistry, Maharaja Jitendra Narayan Medical College & Hospital, Cooch Behar; 4 Assistant Professor, Dept. of Orthodontics and Dentofacial Orthopaedics, Dr. R. Ahmed Dental College & Hospital, Kolkata

## Abstract

**Background:**

The aim of this study was to compare Bolton’s Overall and Anterior ratio in different types of malocclusions.

**Material and Methods:**

105 study cast were taken from the patients undergoing orthodontic treatment and they were divided into three classes based on molar relation, i.e. Angle’s Class I (n = 35), Angle’s Class II (n = 35) and Angle’s Class III (n = 35) according to the Angle’s classification of malocclusion. The mesio-distal widths of all maxillary and mandibular teeth from right first molar to left first molar were measured with a digital calliper. The findings were then used to calculate the anterior and total Bolton’s ratios. Statistical analysis was done using ANOVA and Tukey’s test.

**Results:**

The mean total ratio for the whole sample was 91.4±3.33, which is very close to Bolton’s proposed ideal ratio. The anterior ratio for the whole sample was found to be 77.4±9.02, which is also similar Bolton’s proposed ideal ratio. In Class I patients, the mean total ratio calculated was 91.2 ± 2.69, that is close to Bolton’s proposed ideal ration and the mean anterior ratio calculated was 79.2±3.78, which is greater than Bolton’s proposed ratio that signify increased mesio-distal width in mandibular anterior segment. The total mean ratio calculated for Class II was 91.1 ± 3.44) and for Class III patients was 92±3.77. The anterior mean ratio for class III patients was 78.6±5.24 and for Class II patients was 76.8± 5.28 which indicates higher anterior ration for class III patients and lower anterior ratio for class II patients compared to Bolton’s ideal ratio that signifies mandibular anterior tooth material excess in Class III patients and maxillary anterior tooth material excess in class II cases.

**Conclusions:**

In this study population there was increased mesio-distal width in mandibular anterior segment in patients with Class I and Class III malocclusion. Also, there is maxillary anterior tooth material excess in Class II patients.

** Key words:**Angle’s classification, Anterior ratio , Bolton’s Ratio , Inter-arch relationship, Overall ratio.

## Introduction

Tooth size discrepancy (TSD) refers to the difference in size between individual teeth (1). An ideal occlusion requires teeth in both arches to be proportionate in size. However, substantial TSDs can prohibit an ideal occlusion after orthodontic treatment.

In 1958, Bolton presented tooth size analysis and determined the anterior segment ratio (77.2 ± 0.22%) and total arch ratio (91.3 ± 0.26%). Bolton’s ratio is considered the seventh “key” to achieving perfect occlusion (2). TSDs affect all teeth except permanent second and third molars. Anterior TSDs affect six teeth from the left canine to the right side.

The prevalence of TSDs among orthodontic patients ranges from 4% to 11% (3,4) Orthodontic patients show a prevalence of anterior TSDs ranging from 17% to 31%, similar to non-orthodontic patients (20.5%) (5,6). TSDs are more prevalent in Class II division 1 malocclusions (7) and Class III malocclusions (8). Studies indicate that women have smaller tooth size ratios than men, however the differences are not statistically significant (0.6-1.0%). According to Smith *et al*. (9), black people had the highest total tooth size ratio (93.4%), followed by Hispanics (92.3%) and whites (91.2%).

Measuring the mesio-distal breadth of teeth can be done with a fine-pointed calliper, digital calliper, or photocopies of casts with callipers and dividers (10,11).

Present study was aimed to calculate the overall and anterior Bolton’s ratios in different malocclusion groups of patients in population of West Bengal applying for orthodontic treatment and to compare them with Bolton’s standards.

## Material and Methods

This retrospective study was conducted on the pre-treatment records obtained from the Department of Orthodontics and Dentofacial Orthopaedics at Government Dental College and Hospital, West Bengal. About 105 patients records (53 males and 52 females), who had consulted between Feb 2024 to Jan 2025 were included. The study was approved by the ethical committee of the institution (IEC no.-Ortho/84). All the patients were 12-16 years of age who had permanent dentition. All the 105 patients were divided into three groups based on the Angle’s classification of malocclusion, which is evaluated using their pre-treatment study models, photographs and clinical case records.

Inclusion criteria were:

• Presence of all permanent teeth from first molar to first molar in both arches.

• No caries or extensive restorations on any of the teeth.

Exclusion criteria were:

• Presence of any dental anomaly in morphology of teeth namely mesiodens, taurodontism, etc.

• Presence of deciduous, missing teeth.

• Prior history of orthodontic treatment.

Based on the results of an exclusive pilot study conducted due to lack of previous research on the present study population, sample size estimation was done using G* Power software (Version 3.1.9.7, University Duesseldorf, Germany). The minimum total sample size required was calculated as 105 (N=105) with 35 samples per group (n=35), based on an effect size of 0.31, a power of 80%, and a type I error rate of 0.05.

After orthodontic study models are made, sum of the mesiodistal widths of the maxillary and mandibular teeth that is measured with digital calliper. 105 out of 270 study casts were selected based upon the inclusion and exclusion criteria ( Table 1). Study casts were then seperated according to Class I (n = 35), Angle’s Class II (n = 35) and Angle’s Class III (n = 35) based on Angle’s classification of malocclusion.

-Statistical analysis

The collected data was tabulated in a spreadsheet using Microsoft Excel 2019 and then statistical analysis was carried out using the GraphPad Prism for Windows, Version 9.5 (GraphPad Software, La Jolla California USA). A Shapiro-Wilk’s test and a visual inspection of the histograms, normal Q-Q plots, and box plots showed that the collected data were approximately normally distributed for all the groups. Descriptive statistics were used to report the quantitative variables were reported in terms of mean (central tendency) and standard deviation (measures of dispersion). Comparisons were carried out through one-way Analysis of Variance (ANOVA) with the post-hoc Tukey’s HSD test to analyze the total Bolton’s ratios and anterior Bolton’s ratios between the three classes of malocclusion. One-sample t-test was employed to compare the calculated total and anterior Bolton’s ratios with Bolton’s proposed norms respectively. The *P* value of ≤0.05 was considered the significance level.

## Results

The Total Overall Bolton’s Ratio was found to be 91.4(3.33). Among the classes of malocclusion, the order was as follows Class III [92(3.77)]>Class I [91.2(2.69)]>Class II [91.1(3.44)]. Comparisons were performed using One-way ANOVA Test and it revealed no significant differences between the three malocclusion classes and the total overall Bolton’s ratio [F (3,206) =0.52, *P*=0.67] ( Table 2).

The Total Anterior Bolton’s Ratio was found to be 77.34(9.02). Among the classes of malocclusion, the order was as follows Class I [79.2(3.78)]>Class III [78.6(5.24)]>Class II [76.8(5.28)]. Comparisons were performed using One-way ANOVA Test and it revealed no significant differences between the three malocclusion classes and the total anterior Bolton’s ratio [F (3,206) =0.93, *P*=0.43], ( Table 3).

It was observed that the proposed Overall Bolton’s ratio was less than the calculated Class III ratio and the mean total ratio for the present study and greater than the calculated Class I and Class II ratio. When compared with the one-sample t-test, no statistically significant difference was found for each of the malocclusion classes (*P*>0.05), ( Table 4).

## Discussion

Disparities in tooth size are thought to be a significant determinant in appropriate orthodontic finishing, particularly in the anterior portion. The proper proportion of tooth material in the maxillary and mandibular arches is crucial for achieving a good occlusion. In order to help clinicians, finish in “excellent occlusion” with the correct overbite and overjet, Bolton’s analysis offers a rapid diagnostic tool. In Bolton’s analysis, by measuring the greatest mesio-distal width of each permanent tooth, including all the teeth from the 1st left to the 1st right permanent molar a ratio of 91.3% ±1.91 was found. When only the six anterior teeth of the arch were evaluated, the ratio was 77.2%±1.65.

As per Bolton’s study, patients with the means of anterior and total tooth size ratio above or below 2% of the values established in his research, should be classified as having Tooth size discrepancy.11 The mean ± SD for overall and anterior tooth size discrepancy ratios in the present sample were 91.4±3.33 and 77.4±9.02, ( Table 1) respectively, similar to the study done by Dr Bolton’s himself on white americans (12).

Bolton suggested that a discrepancy greater than 1 SD may create clinical problems. Dr. Crosby and Alexander, 1989; Freeman *et al*., 1996; Santoro *et al*., 2000 (13) define a clinically significant ratio as 2 SD outside Bolton’s mean. Proffit *et al*. (2007) stated that a tooth width discrepancy larger than 1.5 mm creates problems that should be considered in the treatment plan.

In the present study, a comparison of Bolton’s overall and anterior ratio was made between in Class I, II and III patients on study cast based on Angle’s classification of malocclusion (14). The mean total ratio for the whole sample was 91.4±3.33 which is very close to Bolton’s proposed ideal ratio. The anterior ratio for the whole sample was found to be 77.4±9.02, which is also similar Bolton’s proposed ideal ratio ( Table 1).

In Class I patients, the mean total ratio calculated was 91.2 ± 2.69, that is close to Bolton’s proposed ideal ration and the mean anterior ratio calculated was 79.2±3.78, ( Table 1) which is greater than Bolton’s proposed ratio that signify increased mesio-distal width in mandibular anterior segment.

The total mean ratio calculated for Class II (91.1 ± 3.44) and Class III (92±3.77) patients and the anterior mean ratio for class III patients (78.6±5.24) and Class II patients (76.8± 5.28) which indicates higher and lower anterior ratio compared to Bolton’s ideal ration that signifies mandibular anterior tooth material excess in Class III patients and maxillary anterior tooth material excess in class II cases ( Table 1).

**Figure 1:**
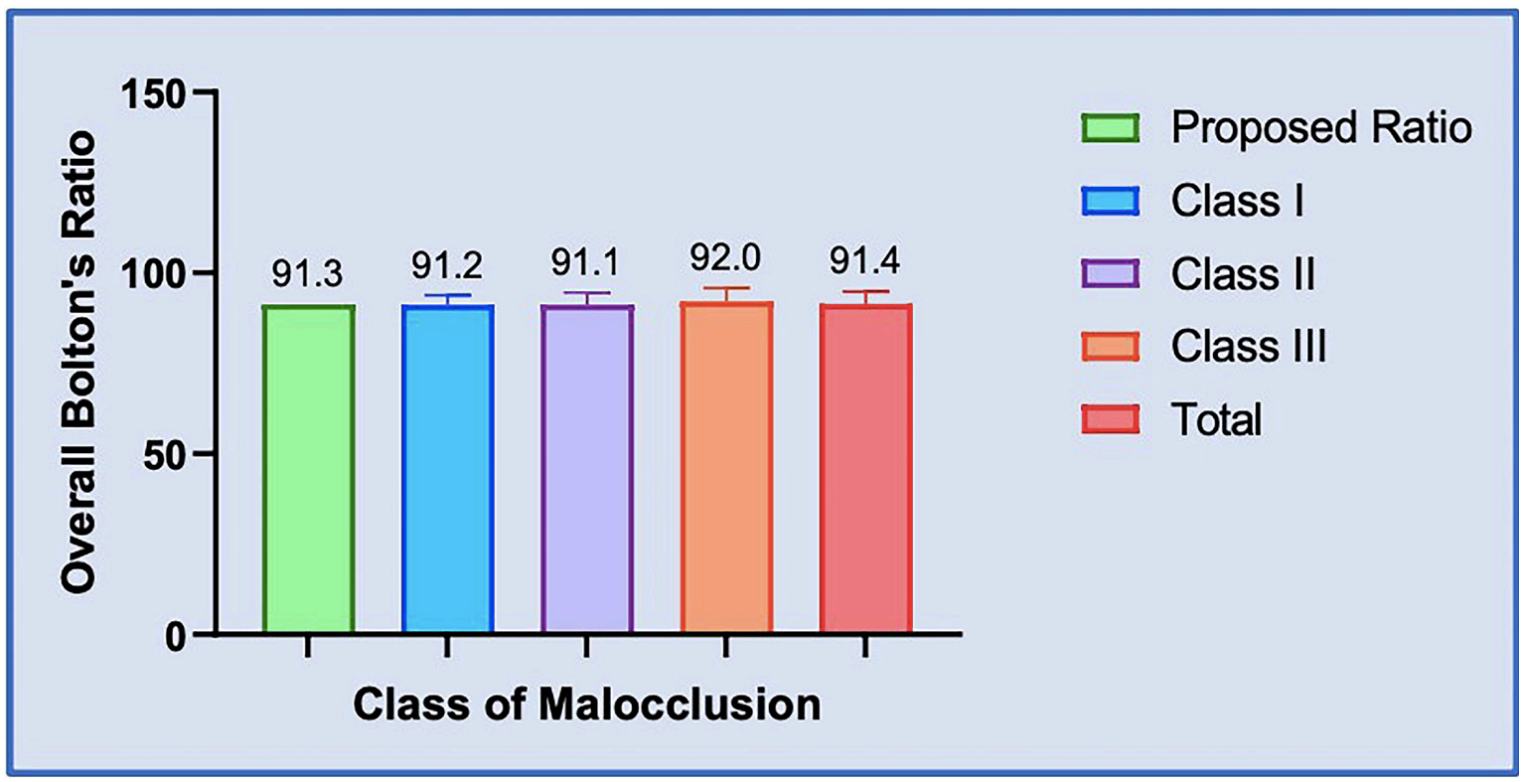
Bar Graph: Comparison of Overall Bolton´s ratio between the three classes of malocclusion and proposed ratio respectively.

**Figure 2:**
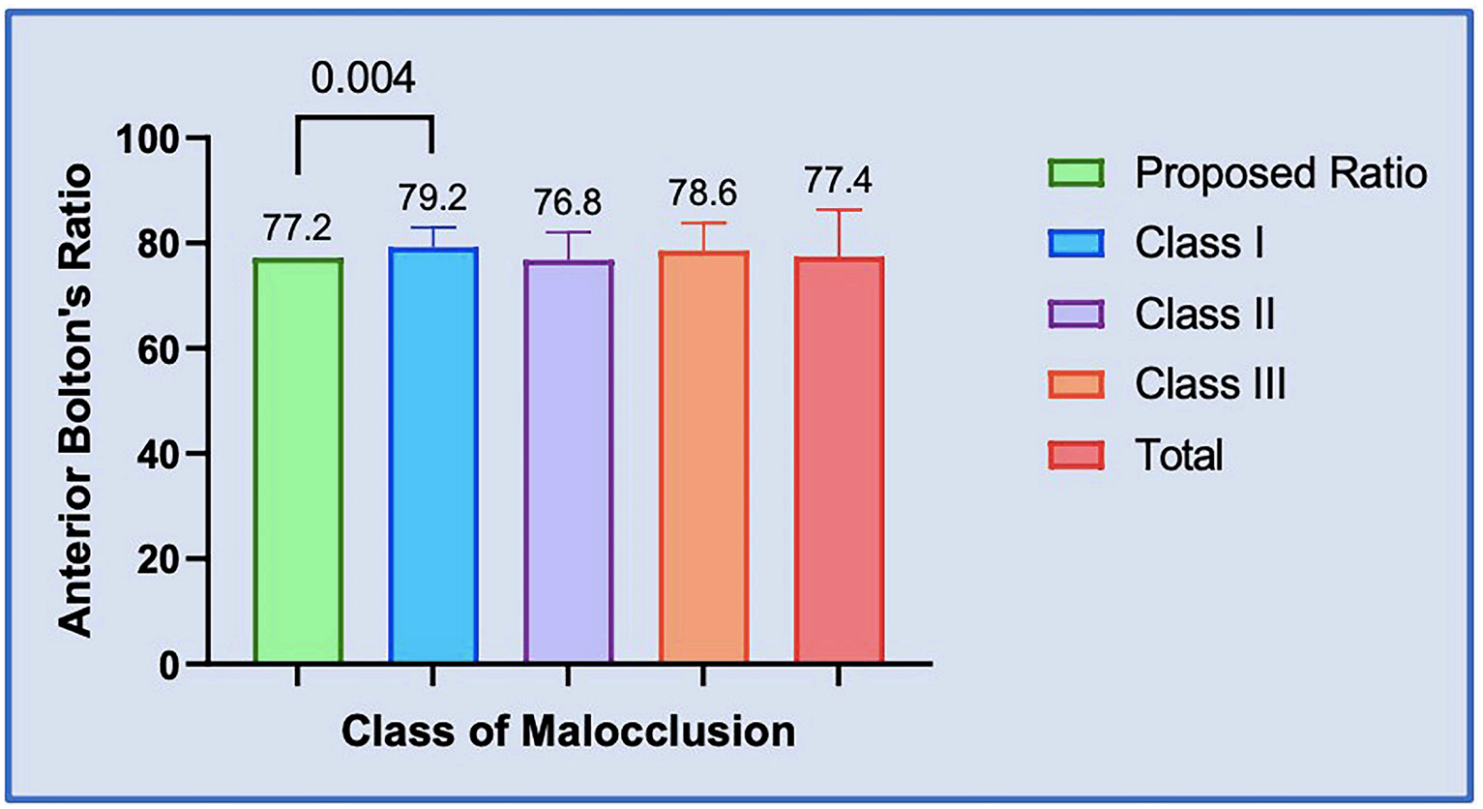
Bar Graph: Comparison of Anterior Bolton´s ratio between the three classes of malocclusion and proposed ratio respectively.

It was observed that the Overall ratio proposed by Dr. Bolton was less than the calculated Class III ratio and the mean total ratio for the present study and greater than the calculated Class I and Class II ratio. When compared with the one-sample t-test, no statistically significant difference was found for each of the malocclusion classes(P>0.05) ( Table 4). Also, the proposed Anterior Bolton’s ratio was less than the calculated Class I, Class III, and the mean total ratio for the present study and greater only than the calculated Class II ratio. When compared with the one-sample t-test, a statistically significant difference was found only between the calculated Class I ratio and the proposed ratio by Dr. Bolton (Adjusted *P* value 0.0004*,  Table 5, Figs. 1,2) indicating mandibular tooth material excess in anterior segment with patients having Angles class I malocclusion. However, the difference was not statistically significant for the others (*P*>0.05,  Table 5).

A greater percentage of patients with discrepancy in anterior ratio than in overall ratio suggests the size of the anterior teeth has, mathematically, less effect on overall ratio (13). Individuals with perfect class I occlusion could be similar for different population but there may be different prevalence of Bolton’s discrepancy in different ethnic groups (15). Patients treated with fixed appliances expect ideal occlusion at the termination of treatment. This may be difficult in Class III malocclusion subjects with an anterior Bolton discrepancy due to a relatively large mandibular tooth size, if not diagnosed before the initiation of treatment.

## Conclusions

1. In our study population there is increased mesio-distal width in mandibular anterior segment in patients with Class I and Class III malocclusion.

2. There is maxillary anterior tooth material excess in Class II patients.

Clinical significance: This study signifies that , to accommodate increased tooth material Angle’s class II cases are having tendency of upper incisor proclination ans class I and class III cases are having tendency of anterior cross bite, bimaxillary protrusion and edge to edge bite.

## Figures and Tables

**Table 1:** Mean (SD) of tooth size ratios for the different malocclusion classes.

Groups	Class I (n=35)	Class II (n=35)	Class III (n=35)	Total (N=105)
Overall Bolton's Ratio	91.2(2.69)	91.1(3.44)	92(3.77)	91.4(3.33)
Anterior Bolton's Ratio	79.2(3.78)	76.8(5.28)	78.6(5.24)	77.4(9.02)

N: Total sample size; n: sample size per group SD: Standard deviation

**Table 2:** Comparison of Overall Bolton’s ratio between the three classes of malocclusion with one-way ANOVA and post-hoc Tukey HSD test.

Groups	Mean Difference	Adjusted P Value
Class I vs. Class II	0.02857	>0.9999 ns
Class I vs. Class III	-0.8429	0.7148 ns
Class I vs. Total	-0.2714	0.9754 ns
Class II vs. Class III	-0.8714	0.6930 ns
Class II vs. Total	-0.3000	0.9673 ns
Class III vs. Total	0.5714	0.8156 ns

ns: not statistically significant (*P*>0.05)

**Table 3:** Comparison of Anterior Bolton’s ratio between the three classes of malocclusion with one-way ANOVA and post-hoc Tukey HSD test.

Groups	Mean Difference	Adjusted P Value
Class I vs. Class II	2.427	0.5004 ns
Class I vs. Class III	0.6400	0.9827 ns
Class I vs. Total	1.841	0.5630 ns
Class II vs. Class III	-1.787	0.7314 ns
Class II vs. Total	-0.5853	0.9761 ns
Class III vs. Total	1.201	0.8308 ns

ns: not statistically significant (*P*>0.05)

**Table 4:** Comparison of Overall Bolton’s ratio between the three classes of malocclusion and proposed ratio respectively with a one-sample t-test.

Calculated Ratio of Groups	Proposed Ratio	Mean Difference	Adjusted P Value
Class I	91.3(0.26)	-0.13	0.77 ns
Class II		-0.16	0.78 ns
Class III		0.7	0.27 ns
Total		0.137	0.67 ns

ns: not statistically significant (*P*>0.05)

**Table 5:** Comparison of Anterior Bolton’s ratio between the three classes of malocclusion and proposed ratio respectively with a one-sample t-test.

Calculated Ratio of Groups	Proposed Ratio	Mean Difference	Adjusted P Value
Class I	77.2(0.22)	2.004	0.004*
Class II		-0.422	0.64 ns
Class III		1.364	0.13 ns
Total		0.16	0.85 ns

ns: not statistically significant (*P*>0.05) *: statistically significant (*P*<0.01)

## Data Availability

The datasets used and/or analyzed during the current study are available from the corresponding author.
